# Selecting Summary Statistics in Approximate Bayesian Computation for Calibrating Stochastic Models

**DOI:** 10.1155/2013/210646

**Published:** 2013-09-01

**Authors:** Tom Burr, Alexei Skurikhin

**Affiliations:** ^1^Statistical Sciences, Los Alamos National Laboratory, Los Alamos, NM 87545, USA; ^2^Space Data Systems, Los Alamos National Laboratory, Los Alamos, NM 87545, USA

## Abstract

Approximate Bayesian computation (ABC) is an approach for using measurement data to calibrate stochastic computer models, which are common in biology applications. ABC is becoming the “go-to” option when the data and/or parameter dimension is large because it relies on user-chosen summary statistics rather than the full data and is therefore computationally feasible. One technical challenge with ABC is that the quality of the approximation to the posterior distribution of model parameters depends on the user-chosen summary statistics. In this paper, the user requirement to choose effective summary statistics in order to accurately estimate the posterior distribution of model parameters is investigated and illustrated by example, using a model and corresponding real data of mitochondrial DNA population dynamics. We show that for some choices of summary statistics, the posterior distribution of model parameters is closely approximated and for other choices of summary statistics, the posterior distribution is not closely approximated. A strategy to choose effective summary statistics is suggested in cases where the stochastic computer model can be run at many trial parameter settings, as in the example.

## 1. Introduction

 To advance knowledge of biological systems, bioinformatics includes a wide range of real and modeled data. For a model with parameters *θ* and data *D*, a key quantity in Bayesian inference is the posterior distribution of model parameters given by Bayes rule as *p*_post_(*θ* | *D*) = *p*(*D* | *θ*)*p*_prior_(*θ*)/*p*(*D*), where *p*_prior_(*θ*) is the probability distribution for *θ* prior to observing data *D*, *p*(*D* | *θ*) is the likelihood, and *p*(*D*) = ∫_*θ*_ *p*(*D* | *θ*)*p*_prior_(*θ*) is the marginal probability of the data, used to normalize the posterior probability *p*_post_(*θ* | *D*) to integrate to 1 [1]. The likelihood *p*(*D* | *θ*) can be regarded as the “data model” for a given value of *θ*. Alternatively, when the data *D* is considered fixed, *p*(*D* | *θ*) is regarded as a function of *θ*, and non-Bayesian methods such as maximum likelihood find the value of *θ* that maximizes *p*(*D* | *θ*) [1]. Regarding notation, note, for example, that *p*(*D* | *θ*) is not the same as *p*(*D*), but to keep the notation simple, we assume the distinction is clear from context.

 In many applications, the data model *p*(*D* | *θ*) is computationally intractable but instead is implemented in a stochastic model (SM), so many realizations from *p*(*D* | *θ*) are available by running the model many times at each of many trial values of *θ*. In a bioinformatics example, [2] considered the classic problem of inferring the time to the most recent common ancestor of a random sample of *n* DNA sequences. The full likelihood of the data *D* involves the branching order and branch lengths, which is known to be computationally intractable because the number of possible branching orders of a sample of *n* DNA sequences grows approximately as *n*!. Therefore, [2] greatly simplified the analysis by replacing *D* with the number of segregating sites (a segregating site is a site that exhibits variation in the DNA character across the sample) *S*_*n*_ in the sample of *n* sequences. The key simplification exploited in [2] is that the distribution of *S*_*n*_ does not depend on the branching order or individual branch lengths, but only on the total length of the phylogenetic tree, which is the sum of all branch lengths. Of course *S*_*n*_ is a summary statistic that has long been of interest in population genetics. But how effective is *S*_*n*_ for estimating the posterior distribution of the time to the most recent common ancestor of the sample? The main point of this paper is to explore the impact of the choice of summary statistic(s) on the quality of the estimated posterior distribution ${\hat{p}}_{\text{post}}(\theta |D)$ when using approximate Bayesian computation (ABC), which is defined in Section 2. We investigate the user requirement to choose good summary statistics to effectively estimate the posterior distribution of model parameters by example, using a model and corresponding real data of mitochondrial DNA population dynamics. 

 In our context, the SM provides the data generation mechanism, so there is no explicit functional form for *p*(*D* | *θ*). Likelihood-free inference dates to at least [3], but the name approximate Bayesian computation (ABC) originated in [4] while referring to an approach to likelihood-free inference methods. Effective values of input parameters for both deterministic and stochastic computer models are typically chosen by some type of comparison to measured data. Parameter estimation for deterministic models is frequently done by running the model at multiple values of the input parameters, constructing an approximator to the model, and using the approximator inside a numerically intense loop that examines many trail values for the input parameters [5–8]. The numerically intense loop is often Markov Chain Monte Carlo (MCMC), which is a method to simulate observations from the posterior distribution of model parameters [1, 9]. Parameter estimation for stochastic models for which an explicit likelihood is not available has been attempted at least once using MCMC with a model approximator [10], but is far more commonly done using ABC. For examples of ABC applied to calibrate SMs, see [11–27] and the many references cited by [11–27]. The example in Section 4 is based on the example in [10], but we use ABC instead of a model approximator inside the MCMC loop.

 The paper is organized as follows. The next section gives background on ABC.  Section 3 describes in more detail the challenge in ABC of choosing effective summary statistics. Section 4 is an example, using a model and corresponding lab data of mitochondrial DNA population dynamics. The example shows that for some choices of summary statistics, the posterior distribution of model parameters is closely approximated and for other choices of summary statistics, the posterior distribution is not closely approximated. A strategy to choose effective summary statistics is suggested in cases where the stochastic computer model can be run at many trial parameter settings, as in the example.

## 2. ABC Background

 Assume that a SM has input parameters *θ* and outputs data *y*_*M*_ = *f*(*y* | *θ*) (*M* for “model”) and that there is corresponding observed real data *y*_obs_. In this section and the remaining sections we either use the conventional notation *y* for data or the informal *D* used in the Introduction, depending on context. We replace the notation for the data generation mechanism *p*(*D* | *θ*) with *f*(*D* | *θ*) to convey the fact that there is no explicit functional form for the likelihood, but only a “black box” SM that outputs data for given values of inputs *θ*. That is, traditionally, the notation *p*(*D* | *θ*) conveys a specific functional form, such as the familiar Gaussian distribution, while the notation *f*(*D* | *θ*) conveys the black box function encoded by the SM.

 The ABC approach uses *y*_obs_ to “calibrate” the SM by choosing effective values for the *θ* parameters. If the SM can be run for many trial values of *θ*, MCMC can be used, where candidate *θ* values are accepted in the chain if the distance *d*(*y*_obs_, *y*_*M*_(*θ*)) between *y*_obs_ and *y*_*M*_(*θ*) is reasonably small. Alternatively, for most applications, and for our focus here, it is necessary to reduce the dimension of *y*_obs_ to a relatively small set of summary statistics *S* and instead accept trial values of *θ* inside the MCMC loop if *d*(*S*(*y*_obs_), *S*(*y*_*M*_(*θ*))) < *T*. For example, *y*_obs_ can be a time series of changes in the proportion of mutant species at various time lags, while *S*(*y*_obs_) could be a scalar count of how often successive differences in *y*_obs_ are larger than a multiple of the measurement error. Most applications of ABC have relied on summary statistics that are chosen on the basis of expert opinion or established practice (such as the number of segregating sites in the example in Section 1) rather than for their role in providing a high quality approximation to the posterior distribution *p*_post_(*θ* | *y*_obs_) [4, 12, 14, 18, 20].

 The goal in nearly all Bayesian inference is to approximate the posterior distribution *p*_post_(*θ* | *y*_obs_) of *θ* given the data *y*_obs_. The ABC approach to do so is to estimate *p*_post_(*θ* | *y*_obs_) = *p*(*y*_obs_ | *θ*)*p*_prior_(*θ*)/*p*(*y*) using the so-called partial posterior distribution *p*_post_(*θ* | *S*_obs_) = *p*(*S*_obs_ | *θ*)*p*_prior_(*θ*)/*p*(*S*_obs_). That is, ABC conditions on the value of the observed summary statistic *S*_obs_ rather than on the actual data *y*_obs_. Because trial values of *θ* are accepted if *d*(*S*(*y*_obs_), *S*(*y*_*M*_(*θ*))) < *T*, an approximation error to the partial posterior distribution arises that several ABC options attempt to mitigate. Such options involve weighting the accepted *θ* values by the actual distance *d*(*S*(*y*_obs_), *S*(*y*_*M*_(*θ*))) [13].

ABC was developed to calibrate a model using summary statistics, but ABC has the potential to choose between candidate models, say models *M*_1_ and *M*_2_. When analytical likelihoods are available, one typically evaluates *P*(*M* | *y*_obs_) using the likelihoods *f*_1_(*y*_obs_) and *f*_2_(*y*_obs_) and the prior probabilities of the models *M*_1_ and *M*_2_. Bayesian model selection is a large topic [1, 4, 12, 21], and it is currently used in calibrating deterministic models using field data [5–8]. Using Bayes rule, *P*(*M*_1_ | *y*_obs_) = (*P*(*y*_obs_ | *M*_1_)*P*(*M*_1_))/*P*(*y*_obs_) and *P*(*y*_obs_ | *M*_1_) are the marginal likelihood for model *M*_1_, defined as *P*(*y*_obs_ | *M*_1_) = ∫*P*(*y*_obs_ | *θ*, *M*_1_)*p*_prior_(*θ*)*dθ*. In model selection to decide between *M*_1_ and *M*_2_, the prior probabilities *P*(*M*_1_) and *P*(*M*_2_) must also be specified so that *P*(*M*_1_ | *y*_obs_) can be compared to *P*(*M*_2_ | *y*_obs_) [1, 21]. The analogous concept in the case of stochastic models is still the posterior distribution *P*(*M*_1_ | *y*_obs_) or *P*(*M*_2_ | *y*_obs_), but summary statistics are used to approximate *P*(*M*_1_ | *y*_obs_) and *P*(*M*_2_ | *y*_obs_). Applications papers have extended ABC to include an option to choose among candidate models that includes different models with possibly different numbers of parameters in a solution space that is explored by simulation [4, 12, 21]. However, the approximation quality of ABC with or without model selection is a subject of ongoing research [18–21].

 ABC is compelling, when the data and/or parameter dimension is large, and is becoming the “go-to” option for many application areas, particularly whenever the likelihood involves summing probabilities over many unobserved states such as genealogies in biology [2], applications in epidemiology [22], astronomy, and cosmology [23]. However, challenges remain in ensuring that ABC leads to reasonable approximation to the full posterior distribution of SM parameters *θ*.

## 3. Choosing Summary Statistics for ABC

 To obtain samples from the approximate posterior distribution for candidate models and model parameters, ABC invokes MCMC [1, 9, 17] with summary statistics such as moments of the observed data to those in the simulated data to decide whether to accept each candidate model and set of parameter values inside the MCMC loop. Note that in cases where the likelihood (the probability density function, pdf, viewed as a function of the parameters values) is known except for a normalizing constant, MCMC has been the main option for numerical Bayesian inference since the 1990s [1]. The main challenges with MCMC using a known likelihood function are that efficient sampling methods are sometimes needed to choose candidate parameter values, and in all cases the burden is on the user to check whether the MCMC is actually converging to the correct full posterior distribution. Because ABC simply accepts trial values of the parameters provided *d*(*S*(*y*_obs_), *S*(*y*_*M*_(*θ*))) < *T*, a common version of ABC uses a very specialized form of MCMC that is called the “rejection” method. Other ABC versions are under investigation [17].

ABC typically consists of three steps: (1) sample from the prior distribution of parameter values *p*_prior_(*θ*); (2) simulate data for each simulated value of *θ*; (3) accept a fraction of the samples prior values in (1) by checking whether the summary statistics computed from the data in (2) satisfy *d*(*S*(*y*_obs_), *S*(*y*_*M*_(*θ*))) < *T*. If desired, adjust the accepted *θ* values on the basis of the actual *d*(*S*(*y*_obs_), *S*(*y*_*M*_(*θ*))) value. Despite the simplicity of ABC, open questions remain regarding to what extent ABC achieves its goal of approximating the full posterior probability. ABC has been shown to work well in some cases [19], but it has also proven not to work well in other cases [21]. There are open questions for ABC regarding the choice of summary statistics [18–21], whether model selection via ABC is viable (meaning that the user can know whether the estimation quality of the full posterior distribution is adequate to successfully compare candidate models [21]), and regarding error bounds for the estimated posterior distribution. Approximate error bounds are possible by simulation using auxiliary simulations such as in Section 4 and [20]. 

 ABC requires the user to make three choices: the summary statistics, the threshold *T*, and the distance measure *d*. This paper's focus is on the user's choice of summary statistics. Recall from the Introduction that in many applications of ABC, the user chooses summary statistics such as the number of segregating sites in a random sample of *n* DNA sequences simply because such a statistic is heavily used in the application area without considering whether the chosen summary statistic renders the partial posterior to be a good approximation to the full posterior. 

 A few recent papers have considered summary statistic selection from the viewpoint of aiming for better inference or better approximation to the full posterior probability [18–21]. ABC makes two approximation steps. First, the full posterior probability is estimated by the partial posterior probability. Second, the partial posterior probability is itself estimated. Recall from Section 2 that some versions of ABC include options to improve the quality of the partial posterior approximation, such as weighting the accepted parameter values in the MCMC [12, 13]. 

 To improve the choice of which partial posterior approximation to use, the notion of approximate statistical sufficiency can be invoked to try to choose more effective summary statistics [18]. Suppose there is a list of *k* candidate summary statistics {*S*_1_, *S*_2_,…, *S*_*k*_}. A user then wonders whether adding candidate statistic *S*_*k*+1_ would improve the approximation of the full posterior *p*_post_(*θ* | *y*_obs_). In [18], ABC must be performed on {*S*_1_, *S*_2_,…, *S*_*k*_} and then on {*S*_1_, *S*_2_,…, *S*_*k*+1_}. If the calculated ratio $R_{k}(\theta )={\hat{p}}_{\text{post}}(\theta |S_{1},S_{2},\ldots S_{k+1})/{\hat{p}}_{\text{post}}(\theta |S_{1},S_{2},\ldots S_{k})$ is statistically significantly different from 1, include candidate statistic *S*_*k*+1_. The framework in [18] is therefore the same framework as for variable selection in fitting any response, so the full arsenal of possibilities in modern data mining is possible. To date, only relatively simple variable selection that is sensitive to the order with which candidate summary statistics are presented has been assessed, only in the few examples in [18]. In [18], the procedure to decide whether *R*_*k*_(*θ*) is statistically significantly different from 1 involves an auxiliary simulation and calculating the maximum and minimum values of *R*_*k*_(*θ*) on a user-chosen grid of *θ* values. An even more computationally demanding option to decide whether *R*_*k*_(*θ*) is statistically significantly different from 1 could invoke some type of density estimation. The simulation approach in [18] makes no judgment whether including candidate summary statistic *S*_*k*+1_ leads to a better approximation. Instead, the simulation approach aims to infer whether including *S*_*k*+1_ has a significant impact on the estimated partial posterior distribution.

Alternatively, to choose effective summary statistics [19] aims to make the selection of summary statistics more “automatic” and less user dependent by requiring the user to run pilot simulations of the model. However, the examples in [19] illustrate the potential for poor ABC performance because the three ABC choices that lead to best performance were shown to vary across examples. The suggested strategy in [19] requires pilot runs of the model in order to improve the user choices, particularly of the summary statistics. The pilot runs require a set of input parameter values *θ*′ to generate data that is similar to the real data *y*_obs_. The goal is then for many realizations of the data from parameter values *θ*′ to help the user choose summary statistics. Specifically, for ABC to lead to good estimation of *θ*, [19] shows that the estimated posterior means of the parameters based on the pilot runs are effective summary statistics. There are several options described in [19] to estimate the posterior means of model parameters. The simplest one to describe is to fit in turn each individual parameter in *θ* using some type of data transformation, such as the actual data and moments of the data. The fitted coefficients from the fit are obtained from the pilot simulation runs and can then be used in subsequent runs to estimate the parameter means. Reference [20] also aimed for better estimation of *θ* and also used auxiliary simulations, but, unlike [19], summary statistics were pursued that minimized the entropy (uncertainty) of the estimated full posterior probability. Of course the user might have other criteria, such as for ABC to lead to a good estimation of the full posterior for *θ* as in our example in Section 4 which also relies on auxiliary simulations.

 To summarize this section, the choice of summary statistics is very important if the partial posterior *p*_post_(*θ* | *S*_obs_) obtained using ABC is to provide an adequate approximation to the full posterior *p*_post_(*θ* | *y*_obs_). A few publications have begun to address the issue of summary statistic selection [18–21]. And, a debate has begun to what extent the partial posterior *p*_post_(*θ* | *S*_obs_) obtained using ABC is adequate for model selection [21]. Again, summary statistic selection is an important aspect of ABC's ability to provide adequate model selection capability.

## 4. Example: Mitochondrial DNA Population Dynamics Model

 This section presents an example for which the stochastic computer model is relatively simple so we can generate many observations from the model.

### 4.1. Example

 Neuronal loss in the substantia nigra region of the human brain is associated with Parkinson's disease [10]. Deletion mutations in the mitochondrial DNA (mtDNA) in the substantia nigra region are observed to accumulate with age. A deletion mutation converts a healthy copy of mtDNA to the mutant (unhealthy) variant. The number of mutant copies in cases with Parkinson's disease tends to be higher than in controls without Parkinson's disease. The role that mtDNA deletions play in neuronal loss is not yet fully understood, so better understanding of how mtDNA deletions accumulate is an area of active research. Reference [10] used a simple stochastic model that allowed for any of five reactions, occurring at rates to be estimated. The five reactions are mutation, synthesis, degradation, mutant synthesis, and mutant degradation.

 Let *Y*_1_ denote the number of healthy (1) mtDNA copies and *Y*_2_ denote the number of unhealthy (2) (mutant) mtDNA copies. Following [10] we assume the following five reactions are possible, with the reaction rates as specified. The lower case *y*_1_ and *y*_2_ refer to an individual cell of type 1 or 2. So, for example, reaction *R*_1_ below depicts a single cell of type 1 mutating to type 2 at a rate *c*_1_ *Y*_1_.

  
*R*_1_ : *y*_1_ → *y*_2_ at rate *c*_1_ *Y*_1_
  
*R*_2_ : *y*_1_ → 2*y*_1_ at rate *c*_2_ *Y*_1_/(*Y*_1_ + *Y*_2_) = 1000 *c*_3_ *Y*_1_/(*Y*_1_ + *Y*_2_)
  
*R*_3_ : *y*_1_ → 0 at rate *c*_3_ *Y*_1_
  
*R*_4_ : *y*_2_ → 2*y*_2_ at rate *c*_4_ *Y*_2_/(*Y*_1_ + *Y*_2_) = 1000 *c*_3_ *Y*_2_/(*Y*_1_ + *Y*_2_)
  
*R*_5_ : *y*_2_ → 0 at rate *c*_3_ *Y*_2_.

**Figure 1:**
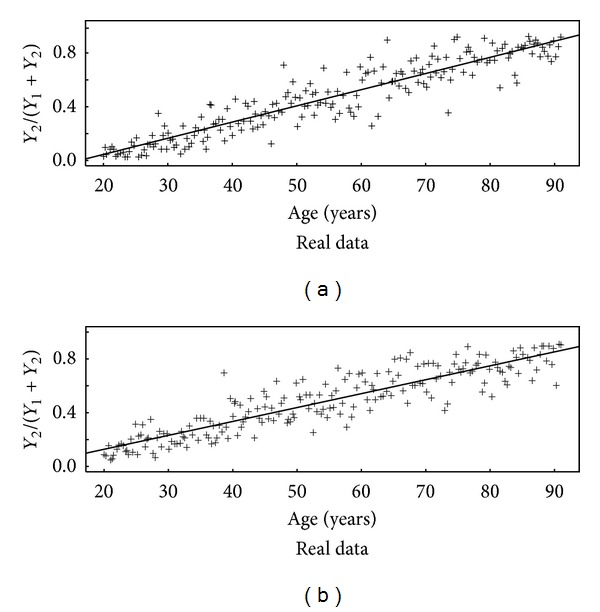
Real (aggregated over patients and measurements of the same patient at the same age) from [10] (a) and corresponding simulated data from the SM (b).

The time between reactions is assumed to have an exponential distribution. The sum of the five rates is the total reaction rate, which determines exponential parameter (the average time between reactions). Given that a reaction occurs at a specific time, the relative rates determine the probabilities with which the five reactions occur. To model the harmful effects of mutation from type 1 to type 2 cells, it is assumed that a cell dies if its proportion of mtDNA deletions *Y*_2_/(*Y*_1_ + *Y*_2_) > *τ* for some lethal threshold *τ*. This simple model can be simulated from exactly using Gillespie's discrete event simulation [28]. Reference [10] gives more information, including information about measurement error models. To focus on summary statistic selection, we simplify the measurement assumptions and measurement error model used in [10] and assume that measurements of {*Y*_1_, *Y*_2_} are available at a sequence of times {*t*_1_, *t*_2_,…, *t*_*n*_}. The real measurement data will be assumed to be of this form, although the measurement details and number of neurons sampled multiple times from each of 15 patients of varying ages make the measurement process used in [10] somewhat more complicated. In particular, the model in [10] did not include a between-patient factor, so we simplified the data by aggregating the data over patients and measurements of the same patient at the same age.  Figure 1 plots the aggregated real data from Figure 1 of [10] and from one realization of simulated data assuming that cells are measured each day.

Note that rates *c*_2_ and *c*_4_ are assumed to be known multiples of rate *c*_3_, so the inference goal is to estimate {*c*_1_, *c*_3_, *τ*}. A range of possible values for each of {*c*_1_, *c*_3_, *τ*} was based in [10] on previous investigations. The prior range for *c*_1_ was 10^−6^ to 10^−3^ per day, for *c*_3_ was 3 × 10^−5^ to 10^−3^ per day, and for *τ* was 0.5 to 1. All three of these parameters are of interest not just as model calibration parameters, but for their physical implications. For example, it is not yet known whether neurons can survive with very high levels of mtDNA deletions. As with any Bayesian analysis, an evaluation of the sensitivity to the prior distribution for the model parameters should be included, particularly for informative priors. In our example, we used informative priors, uniform over the accepted ranges. A separate simulation confirmed that the estimated posterior is sensitive to the assumed parameter range. An example comparison using two ranges for the uniform priors is given in Section 4.4.

The approach in [10] to estimate {*c*_1_, *c*_3_, *τ*} is based on approximating the computer model using a Gaussian Process, which is a common approach in calibrating deterministic computer models. Reference [10] mentions the possibility of estimating {*c*_1_, *c*_3_, *τ*} using ABC. Future work will compare such options. Here, we focus on better understanding of the effect of summary statistic selection on ABC performance.

### 4.2. ABC Approach

 Recall that we assume measurements of {*Y*_1_, *Y*_2_} are available at a sequence of times {*t*_1_, *t*_2_,…, *t*_*n*_}. The real data we use is in Table 1 of [10], which for simplicity we aggregate over subjects and measurements within subjects to the data shown in Figure 1 (see Section 4.4). Note that the real data is observed much less frequently than once per simulated time step which is one day in our simulation. For completeness, we first assume real data is available once per day and then assume real data is available much less frequently, such as in Figure 1(a).

 In any implementation of ABC the user must specify the distance measure, the acceptance threshold *T*, and the summary statistics. In addition, the user chooses the number of model runs at each value of the parameter vector and the number of values of the parameter vector *θ* = {*c*_1_, *c*_3_, *τ*} presented to the ABC algorithm. The statistical programming language R is among the good choices for ABC implementation; here we use the abc function in the abctools package for R [29]. The abc function also requires the user to decide whether to work with transformed parameter values and to select a method to improve estimation of the partial posterior by adjusting the accepted *θ* values according to the distance between the summary statistics and the observed summary statistics [4, 13]. The default method is the “unadjusted” method which accepts all *θ* values corresponding to *d*(*S*(*y*_obs_), *S*(*y*_*M*_(*θ*))) < *T* without any weighting. Results given in Section 4.4 are for the unadjusted option and for the option that adjusts accepted *θ* values.

### 4.3. Simulation Approach

 Our goal for this mDNA example is to illustrate an approach to making good choices for the summary statistics when the user wants the estimated partial posterior distribution for *θ* to be well calibrated. Well calibrated in this example context means, for instance, that the true *θ* is contained in approximately 95% of repeated constructions of 95% predictive intervals for *θ*. That is, the actual coverage is very close to the nominal coverage. 

To check our ABC “calibration,” we repeated the following simulation procedure using *n*_rep_ = 1000 replications and recorded how often nominal intervals containing 95%, 90%, 80%, 60%, 50%, 40%, 20%, 10%, and 5% of the estimated posterior probability *p*_post_(*θ* | *S*_obs_) (which serves as an estimate of *p*_post_(*θ* | *y*_obs_)) actually contain the true parameter value for the three parameters in *θ* = {*c*_1_, *c*_3_, *τ*}.

*Simulation Procedure*

*Step A.* Simulate data from the SM at many parameter values *θ* = {*c*_1_, *c*_3_, *τ*}. Specifically,

1. select each of {*c*_1_, *c*_3_, *τ*} from their respective uniform prior distributions (the ranges are given in Section 4.1) for *n*_sim_ = 1000 simulations,
2. for each selected value of {*c*_1_, *c*_3_, *τ*}, simulate up to 100 years of 1-day step sizes of the five reaction rates. If *Y*_2_/(*Y*_1_ + *Y*_2_) > *τ* at any step, terminate. Some variations of ABC will repeatedly simulate in step (2) for the chosen {*c*_1_, *c*_3_, *τ*} values in step (1).

*Step B.* Real data (or simulated, but with the simulated playing the role of real data):

1. Real data: Either use real measurement data *y*_obs_ or mimic one realization of real measurement data by repeating Step A once. Here we use simulated measurement data to mimic real data, so that we can know the true value of *θ* = {*c*_1_, *c*_3_, *τ*}.
2. Using the *n*_sim_ = 1000 simulations from Step A, accept the trial *θ* = {*c*_1_, *c*_3_, *τ*} values from 100 (10%) of the *n*_sim_ = 1000 simulations on the basis of *d*(*S*(*y*_obs_), *S*(*y*_*M*_(*θ*))) in each of the *n*_sim_ = 1000 simulations, resulting in an approximation of the partial posterior probability *p*_post_(*θ* | *S*_obs_) which serves to estimate the full posterior probability *p*_post_(*θ* | *y*_obs_). The accepted trial *θ* values can be used “as is” to approximate *p*_post_(*θ* | *y*_obs_) or adjusted to account for the actual distance *d*(*S*(*y*_obs_), *S*(*y*_*M*_(*θ*))), for example, as in [4, 13].

*Step C*. (1) Use the 100 accepted trial values of in Step B to tally whether the true parameter values are contained within the estimated 95%, 90%, 80%, 60%, 50%, 40%, 20%, 10%, and 5% posterior intervals. 

 This 3-step simulation procedure is repeated for *n*_rep_ = 1000 replications. Following [10], each simulation began with *n* = 1000 cells. We depart slightly from [10] in that each simulation began with *Y*_2_ = 600 mutant cells (rather than 0 mutant cells), so that each run of up to 100 years tended to conclude in a modest number of years from the starting point due to *Y*_2_/(*Y*_1_ + *Y*_2_) exceeding the lethal maximum *τ*, so that run times are shorter; this mimics starting with older subjects, nearly all of which do not live close to 100 years beyond their fictitious starting age defined by having 600 mutant cells at the start of the simulation. Such a choice will impact our inference results, analogous to choosing data ranges in calibration experiments. However, our topic is the choice of summary statistics rather than experimental design for choosing effective data ranges (the data ranges are the subjects' ages in our example).

 Also following [10] we simulated the effects of measurement error, but for simplicity we assumed there was only one measurement method rather than two. To mimic measurement errors due to finite number of observations and the actual measurement process itself, we assume that only 300 of the 1000 cells were observed and that *Y*_1_/(*Y*_1_ + *Y*_2_) fraction was measured with a relative random error standard deviation of 0.20 on the log_10_ scale. These two effects (observing 300 of 1000 and 0.1% relative error standard deviation on log_10_(*Y*_1_/(*Y*_1_ + *Y*_2_)) result in a root mean squared error of approximately 0.13 in the measured relative frequency *Y*_1_/(*Y*_1_ + *Y*_2_) on average across the range of *Y*_1_/(*Y*_1_ + *Y*_2_) values. In comparison, [10] assumes an absolute random error standard deviation of 0.25 on the log_2_ scale. Because two measurement techniques are combined in [10], which complicated the analysis beyond our needs here, we do not attempt to exactly mimic their approach, but only to use reasonable measurement error assumptions for illustration.

#### 4.3.1. The Summary Statistics

 Let *Z*_1_ denote the measured value of *Y*_1_, and *Z*_2_ denote the measured value of *Y*_2_. The first candidate set of summary statistics is the following three: the average rate of change of *Z*_1_/(*Z*_1_ + *Z*_2_), coefficients *b*_1_ and *b*_2_ in a linear model relating the change in *Z*_1_ (the response) to predictors consisting of the current *Z*_1_, and the current ratio *Z*_1_/(*Z*_1_ + *Z*_2_). The second candidate set of summary statistics is the same as the first, but also includes the maximum of the observed ratio *Z*_1_/(*Z*_1_ + *Z*_2_) and the number of steps until cell death. The third candidate set of summary statistics is the same as the first, but also includes coefficients *b*_1_ and *b*_2_ in a linear model relating the change in *Z*_2_ (the response) to predictors consisting of the current *Z*_2_, and the current ratio *Z*_1_/(*Z*_1_ + *Z*_2_). All three candidate sets of summary statistics were computed for sets of simulated data that was observed at each time step (day), and also much less frequently as in the real data. To mimic the real data, we sampled the simulated data at 13 random times over the duration of each simulation. 

 Concerning the choice of summary statistics, these three candidate sets are arbitrary but reasonable statistics that clearly relate to the SM and so are informative for the SM parameters. For example, the average rate of change of *Z*_1_/(*Z*_1_ + *Z*_2_) relates directly to parameter *c*_3_. 

### 4.4. Example Results

Here we present results for three candidate sets of summary statistics. Our strategy involves two criteria. First, retain for consideration any set of summary statistics that leads to a well-calibrated estimate of *p*_post_(*θ* | *y*_obs_) on the basis of the 3-step simulation procedure. Here, the term “well calibrated” means that actual coverage is very close to the nominal coverage. Second, among all sets of such summary statistics, choose the set that has the smallest estimation error for *θ*. The second criterion is similar to that suggested in [19, 20]. The strategy in [18] described in Section 3 to decide whether adding an additional statistic will impact the posterior could of course also be used to confirm that the three candidate sets of summary statistics do lead to meaningfully different estimates of the partial posterior distribution*p*_post_(*θ* | *S*_obs_). 

**Figure 2:**
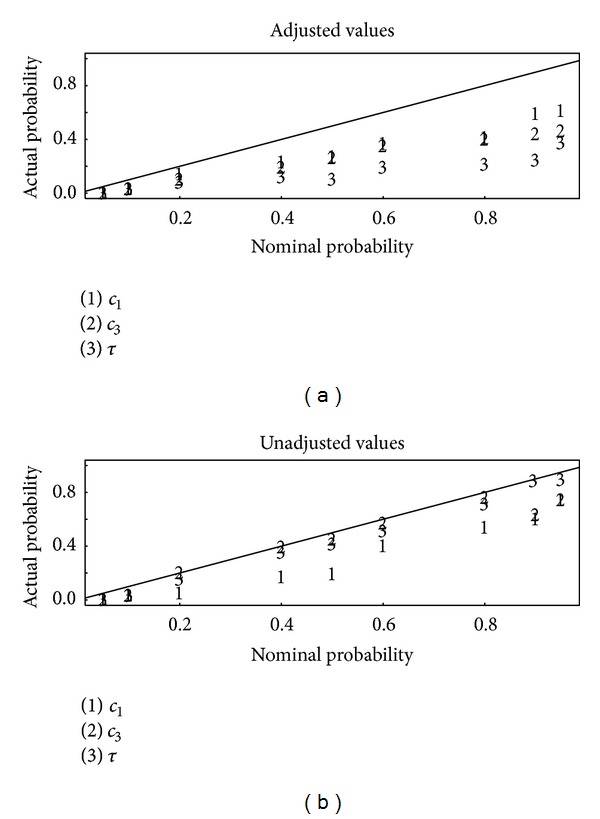
Actual (estimated to within ±0.03 on the basis of 1000 replications of the simulation approach) coverage versus the nominal coverage for 95%, 90%, 80%, 60%, 50%, 40%, 20%, 10%, and 5% posterior intervals for each of the three parameters for each of the three sets of summary statistics, using the ridge-based adjustment (a) in abc or not using any adjustment (b). This plot is based on summary statistics set 1.

**Figure 3:**
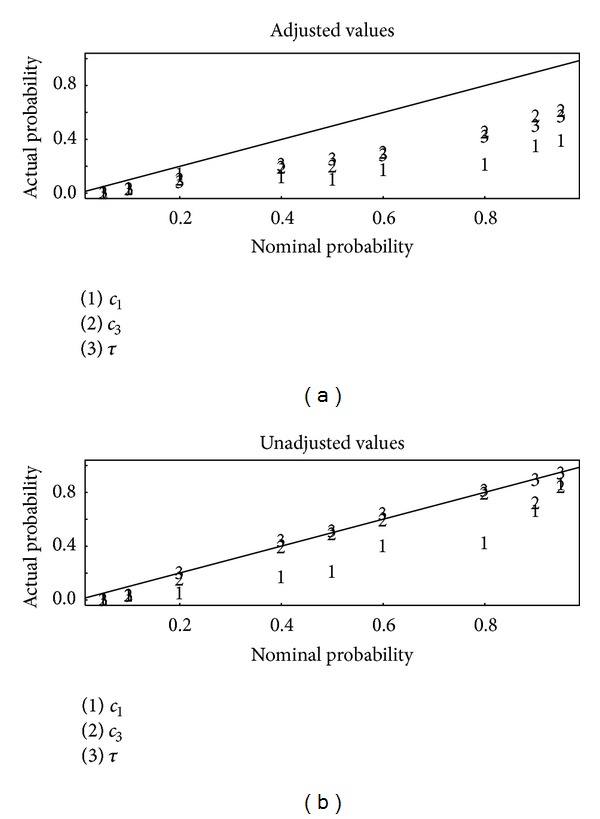
Same as Figure 2, but for summary statistics set 2.

**Figure 4:**
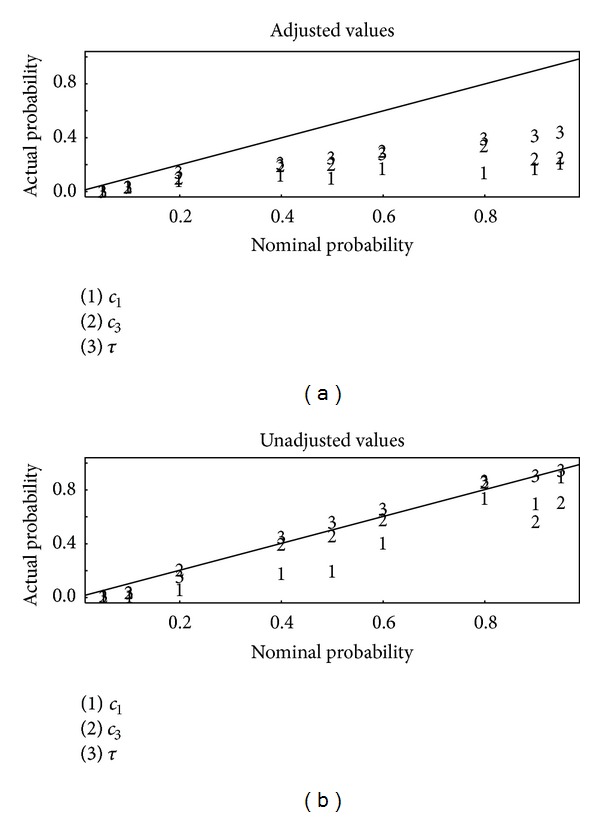
Same as Figure 2, but for summary statistics set 3.

Figure 2 is a plot of the actual (estimated to within ± 0.03 on the basis of 1000 replications of the simulation approach) coverage versus the nominal coverage for 95%, 90%, 80%, 60%, 50%, 40%, 20%, 10%, and 5% posterior intervals for set one of the three sets of summary statistics, using the ridge-based adjustment of the accepted *θ* values in abc or not [4, 13]. Ridge-based adjustment is a form of local ridge regression (a modification of ordinary regression to adjust for collinearity of the predictors) that uses the actual distance *d*(*S*(*y*_obs_), *S*(*y*_*M*_(*θ*))) rather than the simple rejection criterion.  Figure 3 is the same as Figure 2 but for summary statistics set 2.  Figure 4 is the same as Figure 2 but for summary statistics set 3. Notice from Figures 2–4 that the unadjusted values lead to better calibration than the adjusted values, with the actual probabilities being closer to the nominal probabilities. Apparently, although it is reasonable to adjust accepted parameter values by using the actual distance *d*(*S*(*y*_obs_), *S*(*y*_*M*_(*θ*))) [4, 13], whether such adjustment improves the approximation to the posterior depends on the specifics of each data set, including the adequacy of the chosen summary statistics. It is for that reason that available software such as abc allows the user to compute both adjusted or unadjusted *θ* values.

To quantify the results shown in Figures 2–4 we compute the root mean squared error (RMSE) between the observed coverage probability and the nominal coverage probability for the nine posterior intervals (95%, 90%, 80%, 60%, 50%, 40%, 20%, 10%, and 5%) for each parameter estimate for each of the three sets of summary statistics, $\text{RMS}{\text{E}}_{1}=\sqrt{\sum_{i=1}^{n_{\text{rep}}}\sum_{j=1}^{9}{\left(p_{i,\text{observed},j}-p_{i,\text{nominal},j}\right)}^{2}/9n_{\text{rep}}}$. Informally, we can choose the candidate set of summary statistics that has the smallest RMSE_1_. More formally, to determine whether the smallest RMSE_1_ among the three (or any number of) candidate sets of summary statistics is significantly smaller than the second smallest RMSE, we can repeat the entire simulation procedure approximately 100 times and rank the candidate sets of summary statistics on the basis of their RMSE_1_ values across the 100 repetitions of the 3-step simulation. In this example, candidate set 3 has the smallest RMSEs among the three sets of candidate summary statistics. For any set of candidate summary statistics that are acceptable on the basis of RMSE_1_, the second version of the RMSE defined as $\text{RMS}{\text{E}}_{2}=\sqrt{\sum_{i=1}^{n_{\text{rep}}}{\left({\hat{\theta }}_{i}-\theta_{i}\right)}^{2}}/n_{\text{rep}}$ in estimating *c*_1_, *c*_3_, and *τ* should be evaluated. In RMSE_2_, *θ*_*i*_ is either *c*_1_, *c*_3_, or *τ*, and ${\hat{\theta }}_{i}$ is the corresponding estimate. As the corresponding estimate, we use the mean of the corresponding estimated posterior. The two types of RMSEs for the three sets of candidate summary statistics are listed in Table 1.

There is no guarantee that the “best” set of candidate summary statistics will dominate the other choices of summary statistics. For example, summary statistic set 3 is our choice in this example, but it has higher RMSE_2_ for *τ* than the other two choices. As a reviewer has pointed out, such an outcome requires a user choice, and we suggest “majority rule,” meaning that we choose the summary statistic set that has the smallest RMSE_1_ and/or RMSE_2_ (depending on user needs) for the most number of parameters. So, in this case we invoke “majority rule” and choose summary statistic set 3.

 Any application of ABC that does not include a simulation evaluation such as this one or similar ones in [18–21] is incomplete. Somewhat unfortunately, this means that the choice of summary statistics is not truly “automatic," because it relies on intensive simulations in addition to those in standard ABC. However, the choice of summary statistics can be regarded as “objective,” because a similar strategy is a necessary part of a complete ABC application.

**Figure 5:**
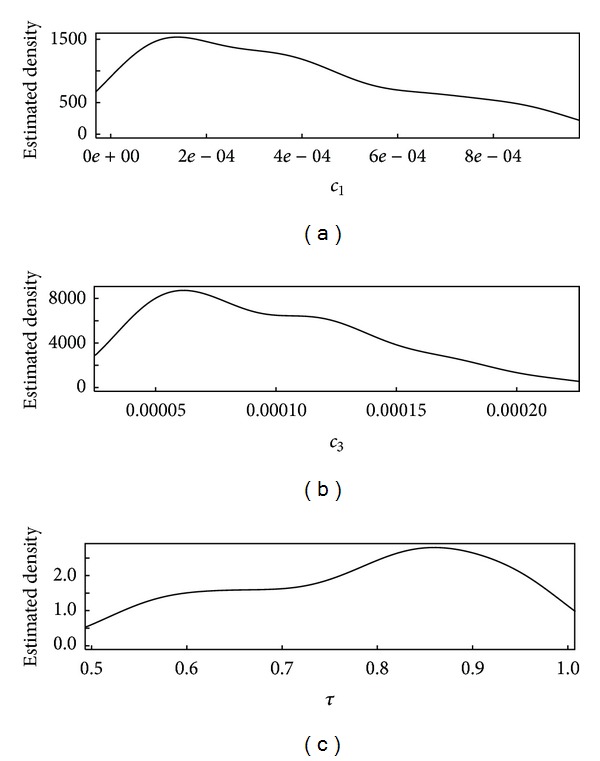
Estimated posterior distribution for *θ* = {*c*_1_, *c*_3_, *τ*} using candidate summary statistics set 3 for the real data, observed only 13 times over multiple years.

 For completeness here, we also use the real data from Table 1 of [10] in place of the simulated data described in the simulation procedure above. Recall from above that the best results (lowest RMSEs) were obtained using candidate summary statistics choice 3 with no adjustment of the accepted *θ* values. However, the real data is observed less frequently than each time step (day), so next we use slightly different summary statistics than described above. Rather than lag-one (one-day) changes, we use the actual times between measurements so that approximate rates of changes can be computed. The resulting posterior for the data in Table 1 of [10] is given in Figure 5 for summary statistic choice 3. Additionally, a second set of simulations was done for simulated data observed only approximately 13 times over the simulation (as in the real data in Figure 1(a)). Again, summary statistic choice 3 had the lowest RMSE_1_ and RMSE_2_ values (uniformly lowest in this case, even for *τ*).

Finally, any Bayesian analysis should address the issue of whether the posterior is sensitive to the prior. For example, in our ABC context, we first used the uniform priors for each parameter as described in Section 4.1, which are the same as those used in [10]. To evaluate sensitivity to the prior, we modified the parameter ranges for *c*_1_ from 10^−6^ to 10^−3^ per day to 10^−7^ to 10^−2^ per day, for c_3_ from 3 × 10^−5^ to 10^−3^ per day to 3 × 10^−4^ to 10^−2^ per day, and for *τ* from 0.50 to 1 to 0.85 to 1. Using summary statistics set 3, the posterior means for {*c*_1_, *c*_3_, *τ*} are 0.0007, 0.0001, and 0.90, respectively, for the original prior ranges and are 0.00007, 0.001, and 0.87, respectively, for the modified prior ranges. These posterior means were each calculated twice using 10^3^ simulations and are repeatable to within the number of digits listed. Therefore, the choice of prior does significantly impact the posterior in our example. Reference [10] discusses the physical consequences of various parameter values, particularly for *τ*. However, there are no widely accepted values for any of the three parameters, so we cannot use accepted parameter values as another check to compare ABC summary statistic choices. Instead, we assess the quality of the ABC-based approximation to the posterior using auxiliary simulation as illustrated in Figures 2–4 comparing predicted to actual coverage probabilities and using RMSEs as in Table 1.

## 5. Summary

ABC is becoming the “go-to” option when the data and/or parameter dimension is large because it relies on user-chosen summary statistics rather than the full data and is therefore computationally feasible. Although ABC is compelling, when the data and/or parameter dimension is large, and is beginning to be used in many application areas, as of 2013, there is no cohesive theory or a consistent strategy for ABC, yet there are many applications in bioinformatics, astronomy, epidemiology, and elsewhere for which a stochastic CM provides an alternative to the likelihood. In addition software to implement ABC is becoming widely available; see [30] for a partial list of currently available ABC software. 

 One technical challenge with ABC is that the quality of the approximation to the posterior distribution of model parameters depends on the user-chosen summary statistics. In this paper, the user requirement to choose effective summary statistics in order to accurately estimate the posterior distribution of model parameters is illustrated by example, using a model and corresponding lab data of mitochondrial DNA population dynamics. The example shows that for some choices of summary statistics, the posterior distribution of model parameters is closely approximated and for other choices of summary statistics, the posterior distribution is not closely approximated. 

 A strategy to choose effective summary statistics is suggested in cases where the stochastic computer model can be run at many trial parameter settings, as in the example. The strategy is to choose the best results from several candidate sets of summary statistics, such as shown in the Results in Figures 2–4. As in [19, 20], auxiliary simulations that produce data having similar summary statistics as the observed data are needed. Then, the best results are defined on the basis of two criteria. First, those summary statistics that lead to the best-calibrated estimated posterior probabilities are identified. Second, among those summary statistics that perform well on the first criterion, those summary statistics that lead to the smallest estimation errors for the parameters *θ* are preferred. The disadvantage of this approach is that reliance on auxiliary simulations to choose summary statistics adds to the computational burden. However, the ABC algorithm is easily parallelized so modern desktop computers are fully adequate for many problems, such as our example. The user might consider using criteria other than those used in Figures 2–4 and in Table 1 to evaluate the posterior distribution. However, we regard those criteria as necessary for adequate approximation to the posterior in this context. 

 Future work will consider the acceptance threshold and variations of ABC such as [17] that mimic standard MCMC sampling rather than using the rejection method with adjustments to the accepted trial *θ* values as in [4, 13]. Also, because real data almost never obey all the assumptions of any model, even the most elaborate stochastic model, some allowance for model bias should be made as that done with deterministic models [6–8]. Finally, a comparison of this ABC approach with the stochastic model approximator approach in [10] would be valuable. 

## Figures and Tables

**Table 1:** The RMSE_1_ and RMSE_2_ values for each of the three sets of candidate summary statistics for *c*_1_, *c*_3_, and *τ* using unadjusted estimates of the respective posterior distribution. The table entries are RMSE_1_ for *c*_1_, *c*_3_, and *τ* in the top line and RMSE_2_ for *c*_1_, *c*_3_, and *τ* in the bottom line. Table entries are based on one set of *n*_rep_ = 1000 replications of the 3-step procedure in Section 4.3 and are repeatable across sets of 1000 replications to the number of digits shown.

Candidate summary statistic	RMSE_1_ for *c*_1_, *c*_3_, and *τ* RMSE_2_ for *c*_1_, *c*_3_, and *τ*
1	RMSE_1_: 0.0002, 0.0002, 0.15 RMSE_2_: 0.19, 0.08, 0.05
2	RMSE_1_: 0.0002, 0.0002, 0.09 RMSE_2_: 0.20, 0.09, 0.08
3	RMSE_1_: 0.0002, 0.0001, 0.09 RMSE_2_: 0.18, 0.07, 0.09
